# Acute kidney injury in severely injured patients admitted to the intensive care unit

**DOI:** 10.1186/s40779-020-00277-1

**Published:** 2020-10-09

**Authors:** Alberto F. García, Ramiro Manzano-Nunez, Juan G. Bayona, Maria P. Naranjo, Dary Neicce Villa, Manuel Moreno, Sebastian Ossa, Juan M. Martinez, Nathalia Martinez, Juan C. Puyana

**Affiliations:** 1 Division of Trauma and Acute Care Surgery, Department of Surgery, Fundación Valle del Lili, Cali, Colombia; 2 Clinical Research Center, Fundación Valle del Lili, Cali, Colombia; 3 Escuela de Medicina y Ciencias de la Salud, Universidad del Rosario, Carrera 98 #18-49, 760001 Bogotá, Colombia; 4 Méderi Hospital Universitario Mayor, Bogotá, Colombia; 5 School of Medicine, Universidad ICESI (ICESI University), Cali, Colombia; 6 Department of Surgery, University of Pittsburgh, Pittsburgh, PA USA

**Keywords:** Rhabdomyolysis, Wounds and injuries, Acute kidney injury, Critical care, Trauma

## Abstract

**Background:**

Our objective was to identify possible associations between clinical and laboratory variables and the risk of developing acute kidney injury (AKI) in severely injured patients admitted to the intensive care unit (ICU) for whom creatine kinase (CK) levels were available.

**Methods:**

For this retrospective observational study, we analyzed adult trauma patients admitted to the ICU from 2011 to 2015 at Fundación Valle del Lili (FVL) University Hospital. Our primary outcome was the incidence of AKI. Multivariate regression analysis was used to assess risk factors for this outcome.

**Results:**

A total of 315 patients were included. The trauma mechanisms were blunt (*n* = 130), penetrating (*n* = 66) and blast (*n* = 44) trauma. The median (interquartile range, IQR) of injury severity score (ISS) was 21 (16–29). AKI developed in 75 patients (23.8%). Multivariate regression analysis revealed that the thoracic abbreviated injury scale (AIS) value (median (IQR) in the AKI group: 3 (0–4)), Acute Physiology and Chronic Health Evaluation (APACHE II) score (median (IQR) in the AKI group: 18 (10–27)), CK greater than 5000 U/L, lactic acid concentration at admission, and dobutamine administration were independently associated with AKI.

**Conclusion:**

We found that age, APACHE II score, thoracic trauma, lactic acidosis, and dobutamine administration were independently associated with AKI. Trauma surgeons need to be aware of the increased odds of AKI if one of these factors is identified during the evaluation and treatment of injured patients.

## Background

Organ dysfunction is a common event among trauma patients who survive the initial insult and undergo damage control resuscitation interventions. Acute kidney injury (AKI), which is one type of organ dysfunction, is often present in severely injured patients, and its occurrence is associated with higher risk-adjusted odds of poor outcomes [1], such as greater critical care resource utilization and higher mortality [2].

Although previous studies have assessed the frequency and factors associated with AKI in trauma patients, the existing literature about the risk factors for AKI after traumatic injuries has its limitations. Published studies have analyzed patients with traumatic and nontraumatic rhabdomyolysis through univariate analyses [3, 4], and variables that can act as effect modifiers, such as hypoperfusion, transfusions, fluid balance and nephrotoxic substances administration, have not been well investigated. In this study, we analyzed a series of trauma patients admitted to the intensive care unit (ICU) in whom creatine kinase (CK) values were available. Our objective was to identify possible associations between clinical and laboratory variables and the risk of developing AKI in severely injured patients admitted to the intensive care unit for whom CK levels were available.

## Methods

### Study design

A retrospective study was performed to determine the incidence and risk factors for AKI after traumatic rhabdomyolysis.

### Data source

For this study, we retrospectively reviewed the clinical records of patients with trauma from 2011 to 2015 at Fundación Valle del Lili (FVL) University Hospital. FVL is a nonprofit private hospital equivalent to a Level I trauma center affiliated with the ICESI University School of Medicine in Cali-Colombia. It is a 511-bed hospital that serves a referral facility for both civilian and military trauma patients from the southwest region of Colombia. The protocol for this study was approved by the Institutional Review Board (Research Ethics Committee) at FVL.

### Patients

Initial screening identified all trauma patients admitted to FVL from January 2011 to December 2015. We included patients older than 17 years of age who were admitted to the ICU and had CK levels available. Patients with chronic kidney disease, grade IV or V renal injury, known pregnancy, moderate or severe burns, or CK elevation not related to trauma were not eligible. To identify nontraumatic causes of CK elevation, we reviewed patients’ past medical history to look for possible causes of nontraumatic CK elevation, such as previously documented rhabdomyolysis, cardiac disease, genetic deficits and use of lipid-lowering drugs.

The primary outcome was the incidence of AKI. There is no consensus on a threshold CK level for diagnosing rhabdomyolysis. However, CK levels are a sensitive indicator of myocyte injury in rhabdomyolysis [5]. Furthermore, elevated CK levels are commonly seen in critically ill patients, and levels of 5000 U/L or higher are related to the occurrence of renal failure in patients with rhabdomyolysis caused by trauma [6, 7]. Therefore, rhabdomyolysis was defined as a CK level greater than 5000 U/L in the absence of a recognized etiology such as cardiac disease or muscular dystrophy. Secondary outcomes included the need for renal replacement therapy (RRT) and mortality. AKI was defined according to the KDIGO definitions [8], and patients with any of the three stages according to the KDIGO criteria were analyzed.

Demographics, trauma characteristics, physiological status, resuscitation and therapeutic strategies were reviewed. The Injury Severity Score was calculated according to the AAST grading of injury severity. The APACHE II score was calculated at day 1 of the ICU admission. RRT was defined as the use of hemodialysis, peritoneal dialysis or continuous RRT. Mortality was defined as death during the hospital stay.

### Statistical analysis

The patients were divided into the AKI group, and the non-AKI group based on final discharge recorded outcomes. The results are summarized with frequencies and percentages for dichotomous and categorical variables and with the mean and standard deviation (SD) or median and interquartile range (IQR) for continuous variables after the analysis of normality with the SK test [9]. Preliminary analysis of the association between potential risk factors and AKI was performed with simple logistic regressions, calculating odds ratios (*OR*) with their corresponding 95% confidence intervals (CI) and *P*-values. Variables with a *P <* 0.1, as well as clinically relevant variables, were entered into a forward stepwise logistic regression model to identify independent risk factors for the development of AKI, keeping into the model those variables with a *P <* 0.1. All analyses were performed with Stata® 12.1 (StataCorp, College Station, TX). The goodness of fit of the final model was evaluated with the Hosmer-Lemeshow technique, and the discriminative ability was evaluated with an ROC curve.

## Results

A total of 315 patients were included. Most of the subjects were male (87.3%), and three-quarters were younger than 41 years (age, median (IQR): 28.0 (22.0–41.0)). The patients were predominantly victims of blunt trauma (*n* = 130) followed by penetrating trauma (*n* = 66) and blast injuries (*n* = 44). Three-quarters of the patients had an ISS greater than 16 (ISS, median (IQR): 21 (16–29)). The median (IQR) of the maximum CK in the first 24 h was 1662 U/L (770–4125). A CK greater than 5000 U/L was present in 60 patients (19.0%). AKI developed in 75 patients (23.8%). We did not find patients with nontraumatic CK elevation.

Patients were divided into two subgroups: those who developed AKI (AKI group) and those who did not (non-AKI group) (Table 1). Patients were similar in demographic characteristics and admission vital signs. Subjects in the AKI group presented significantly higher abdominal and thoracic Abbreviated Injury Scores (AIS). The proportion of patients with trauma to the extremities as well as the extremity AIS were similar among the patients with and without AKI (60.0% vs. 64.0%; extremity AIS, median (IQR): 2 (0–3) vs. 2 (0–3)).

**Table 1:** Patient characteristics, severity scores and resuscitation strategies

Variable	Non-AKI group (*n* = 240)	AKI group (*n* = 75)	Total (*n* = 315)	*P*
Male [*n*(%)]	206 (85.8)	69 (92.0)	275 (87.3)	0.17
Age [years, M (IQR)]	27.0 (22.0–38.5)	33.0 (22.0–45.0)	28.0 (22.0–41.0)	0.15
Trauma mechanism [*n*(%)]				0.01
Penetrating	66 (27.5)	29 (38.7)	95 (30.2)	
Blunt	130 (54.2)	42 (56.0)	172 (54.6)	
Explosion	44 (18.3)	4 (5.3)	48 (15.2)	
SBP [mmHg, M (IQR)]	119 (98–132)	116 (95–138)	117 (98–133)	0.97
BR [breathings/min, M (IQR)]	21 (18–24)	20 (18–26)	21 (18–24)	0.62
Glasgow coma score [M (IQR)]	15 (7–15)	14 (6–15)	15 (7–15)	0.11
AIS head [M (IQR)]	1 (0–4)	0 (0–3)	1 (0–4)	0.18
AIS face [M (IQR)]	0 (0–1)	0 (0–0)	0 (0–1)	0.04
AIS thorax [M (IQR)]	0 (0–3)	3 (0–4)	0 (0–3)	< 0.01
AIS abdomen/pelvis [M (IQR)]	0 (0–2)	0 (0–3)	0 (0–2)	0.01
AIS extremity [M (IQR)]	2 (0–3)	2 (0–3)	2 (0–3)	0.37
AIS external [M (IQR)]	1 (0–1)	1 (0–1)	0 (0–1)	0.05
ISS [M (IQR)]	20 (14–27)	26 (19–34)	21 (16–29)	< 0.01
RTS [M (IQR)]	7.55 (5.97–7.84)	7.55 (5.03–7.84)	7.55 (5.97–7.84)	0.23
APACHE II [M (IQR)]	13 (8.5–18.5)	18 (10–27)	14 (9–21)	< 0.01
CK maximun [M (IQR)]	1924 (726–4940)	2508 (999–10,418)	2081 (794–5785)	0.02
CK > 5000 [*n*(%)]	37 (15.4)	23 (30.7)	60 (19.0)	< 0.01
Initial acid lactic [M (IQR)]	2.98 (1.76–4.56)	4.33 (2.13–6.09)	3.09 (1.77–4.99)	< 0.01
Initial base deficit [M (IQR)]	7.05 (4.5–9.7)	8.7 (5.8–12.4)	7.4 (4.9–10.2)	< 0.01
Fluid balance, first 24 h [Lt., M (IQR)]	1.96 (0.64–3.42)	3.37 (1.57–5.49)	2.19 (0.83–3.81)	< 0.01
Fluid balance 72 h [Lt., M (IQR)]	2.83 (0.27–5.18)	6.28 (3.05–9.48)	3.33 (0.89–6.42)	< 0.01
Packed red blood cells, first 24 h [U, M (IQR)]	0 (0–3)	2 (0–4)	0 (0–4)	< 0.01
Number of patients requiring FFP transfusion [*n*(%)]	52 (21.7)	29 (38.7)	23 (7.3%)	< 0.01
Administration of NaH_2_CO_3_ [*n*(%)]	16 (6.7)	11 (14.7)	27 (8.6)	0.03
Contrast administration [*n*(%)]	149 (62.1)	52 (69.3)	201 (63.8)	0.25
Contrast dose ×100 mg [M (IQR)]	1.0 (0–1.5)	1.0 (0–2.0)	1 (0–2)	0.15
Norephinephrine administration [*n*(%)]	92 (38.3)	45 (60.0)	173 (54.9)	< 0.01
Vasopresina administration [*n*(%)]	30 (12.5)	14 (18.7)	44 (14.0)	0.18
Dobutamine administration [*n*(%)]	9 (3.7)	11 (14.7)	20 (6.3)	< 0.01

*SBP* Systolic blood pressure, *BR* Breathing rate, *AIS* Abbreviate injury score, *ISS* Injury severity score, *RTS* Revised trauma score, *CK* Creatine kinase, *NaH*_*2*_*CO*_*3*_ Sodium bicarbonate

Anatomic and physiologic severity scores were significantly higher in patients with AKI than in patients without AKI (ISS, median (IQR): 26 (19–34) vs. 20 (14–27), *P <* 0.01; RTS, median (IQR): 7.55 (5.03–7.84) vs. 7.55 (5.97–7.84), *P* = 0.23 APACHE II, median (IQR): 18 (10–27) vs. 13 (8.5–18.5); *P <* 0.01).

Individuals in the AKI group were more likely to have significantly higher base deficit and lactate values (*P <* 0.01). The proportion of patients with a CK greater than 5000 U/L was significantly higher in the AKI group (23 of 75 of patients with AKI (30.7%) vs. 37 of 240 of patients without AKI (15.4%); *P <* 0.01).

Among the therapeutic interventions within the first 24 h after admission, a significantly higher number of patients in the AKI group required vasoactive drugs. Norepinephrine was used in 60.0 and 38.3% of patients with and without AKI, respectively (*P <* 0.01). Dobutamine was required in 14.7 and 6.3% of patients with and without AKI, respectively (*P <* 0.01).

In the first 24 h after admission, significantly more patients in the AKI group than in the group of patients without AKI received plasma transfusions (29 of 75 patients with AKI (38.7%) vs. 52 of 240 patients without AKI (21.7%)). Furthermore, patients with AKI received significantly higher volumes of crystalloids in the first 72 h and packed red blood cells transfusions in the first 24 h and were more likely to receive sodium bicarbonate than patients without AKI (Table 1).

**Fig. 1:**
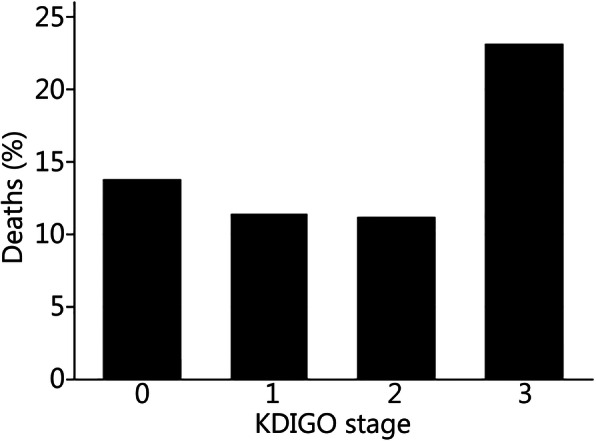
Mortality by acute kidney injury KDIGO stage

There were significant differences in mortality between AKI-KDIGO groups (*P <* 0.001, Fig. 1). The overall mortality was 13%. However, the highest mortality occurred in the patients with KDIGO stage 3 AKI (Fig. 1).

In the univariate analysis, a CK greater than 5000 U/L showed a positive association with AKI risk (*OR* = 2.35, 95%CI 1.29–4.31). After multivariate regression analysis, we found that age, thoracic AIS value (median (IQR) in the AKI group: 3 (0–4)), APACHE II score (median (IQR) in the AKI group: 18 (10–27)), CK greater than 5000 U/L, lactic acid concentration at admission, and dobutamine administration were independently associated with AKI in our study population (Table 2). The final regression model demonstrated adequate calibration (*P* = 0.25) and acceptable discrimination ((area under curve (AUC) = 0.767, 95%CI 0.703–0.832).

**Table 2:** Risk factors associated with acute kidney dysfunction in traumatic rhabdomyolysis

Variables	Simple logistic regressions		Multivariable logistic regression^a^	
	*OR* (95%CI)	*P*	*OR* (95%CI)	*P*
Demographics				
Age (for every 10 years)	1.16 (0.98–1.38)	0.083	1.30 (1.06–1.60)	0.01
Gender				
Male	0.53 (0.2–1.31)	0.167	–	
Female	–			
Trauma mechanism				
Blast	–		0.334 (0.10–1.12)	0.08
Penetrating	3.56 (1.21–10.48)	0.022		
Blunt	4.83 (1.59–14.71)	0.006		
Severity scores				
RTS	0.82 (0.69–0.98)	0.026	–	
ISS	1.04 (1.01–1.06)	0.003	–	
AIS head & neck	0.89 (0.78–1.03)	0.118	–	
AIS face	0.78 (0.59–1.02)	0.068	–	
AIS thorax	1.30 (1.12–1.51)	< 0.001	1.23 (1.04–1.46)	0.02
AIS abdomen/pelvis	1.23 (1.06–1.43)	0.006	–	
AIS extremity	1.09 (0.93–1.29)	0.293	–	
AIS external	0.71 (0.50–1.00)	0.052	–	
APACHE II	1.08 (1.05–1.12)	< 0.01	1.05 (1.01–1.09)	0.03
Biochemical markers				
Initial lactic acid	1.23 (1.11–1.36)	< 0.001	1.17 (1.03–1.33)	0.02
Initial base deficit	1.12 (1.06–1.19)	< 0.01	–	
Higher CK	1.00 (0.99–1.0)	0.12	–	
CK > 5000 U/L	2.35 (1.29–4.31)	< 0.01	2.64 (1.31–5.33)	< 0.001
Resuscitation & nephrotoxic agents				
Fluid balance, first day	1.16 (1.06–1.27)	< 0.01	–	
Fluid balance 72 h	1.20 (1.12–1.28)	< 0.01	–	
Packed red blood cells, first day	1.09 (1.02–1.17)	0.011	–	
FFP transfusion	2.28 (1.31–3.98)	< 0.01	–	
Bicarbonate administration	2.4 (1.96–5.44)	0.035	–	
Contrast administration	1.43 (0.80–2.54)	0.23	–	
Contrast dose ×100 mg	1.32 (1.04–1.67)	0.02	–	
Norepinephrine administration	2.42 (1.42–4.1)	< 0.01	–	
Vasopressin administration	1.59 (0.79–3.19)	0.19	–	
Dobutamine administration	4.37 (1.74–11.0)	< 0.01	3.16 (1.01–9.94)	0.049

^a^Stepwise multivariable logistic regression model with backwards elimination. Variables with *P* value < 0.1 were retained in the model. *OR* Odds ratio, *CI* Confidence interval, *RTS* Revised trauma score, *ISS* Injury severity score, *AIS* Abbreviate injury scale, *CK* Creatine kinase, *FFP* Fresh frozen plasma

## Discussion

This study investigated the risk factors of AKI in trauma patients admitted to the ICU for whom CK results were available. We showed that patients with AKI were more likely to suffer from perfusion derangement, had worse injuries and required more aggressive resuscitation strategies. When adjusting for confounders, we found that increased age, dobutamine administration, the severity of the thoracic injury, the severity of disease (APACHE II), the initial lactic acid level and a CK value greater than 5000 U/L were associated with the occurrence of AKI. Our findings are similar to those reported in a larger multicenter study [10], in which the severity of the trauma, the value of lactate and the presence of hemorrhagic shock were independently associated with the development of AKI. Thus, it is rational to posit that harsh physiological exhaustion driven by the severity of trauma may play a key role in the development of AKI among trauma patients.

We found that increasing age and a CK value greater than 5000 U/L were independently associated with AKI. Age has been uniformly identified in the analysis of risk factors of posttraumatic AKI or multiorgan failure [11–14]. Regarding the role of CK, our results seem to be consistent with previous research, which found that abnormal CK levels are related to renal failure in patients with rhabdomyolysis caused by trauma [3, 6, 7]. For example, Brown et al. [7] analyzed data from 2083 trauma admissions to the intensive care unit and found that a CK value greater than 5000 U/L was independently associated with renal failure. Similarly, Sharp et al. [3] found that an elevated serum CK value was a significant risk factor for AKI. Studies performed in war fighters have confirmed this association and have reported a positive correlation between AKI stages and higher CK values [4, 15].

Once a trauma patient is diagnosed with rhabdomyolysis, his or her prognosis depends heavily on a timely implemented and adequate therapeutic strategy. In addition to rehydration with IV fluids, other interventions such as mannitol and bicarbonate administration have been proposed to prevent AKI [6, 16]. However, the use of bicarbonate to alkalinize the urine remains controversial [17], as no randomized clinical trial supports its use. In this sense, our univariate analysis showed that the administration of sodium bicarbonate was associated with AKI. However, this association could be biased because the administration of bicarbonate could be used for treating metabolic acidosis not to prevent AKI or for a random treatment; our results are consistent with those of Brown et al. [7], who showed that therapy with bicarbonate and mannitol administration was independently associated with renal failure. Furthermore, the administration of bicarbonate in critically ill trauma and nontrauma patients with metabolic acidosis has been shown to paradoxically increase the CO_2_ arterial pressure and thus cellular metabolic acidosis [18, 19]. In contrast, Nielsen et al. [20] showed better outcomes among subjects with traumatic rhabdomyolysis treated with a protocol of forced alkaline diuresis. Beyond bicarbonate administration, it is well known that early IV fluid administration is paramount for improving outcomes among patients with elevated CK levels at risk of AKI [21]; nevertheless, overaggressive fluid therapy has deleterious effects in trauma patients [22]. Although resuscitation strategies with fluid infusion rates of 1 L/h for 2 h after injury and 500 ml/h after 120 min [21, 23] are found in the literature, these recommendations are not supported by randomized controlled trials; thus, we advise caution when infusing large amounts of fluids in patients with AKI secondary to rhabdomyolysis due to trauma.

The current study found that lactic acid elevation and dobutamine administration were independently associated with the development of AKI. Previous studies have revealed an association between impeding hypoperfusion (increased lactate and base deficit) and a greater odds of AKI in trauma patients [3, 24]. For example, Bihorac et al. [24] found that severe trauma patients with a lactate level of 5 mmol/L in the first 24 h had a 25% chance of developing AKI, and this probability increased proportionally with lactate levels. As lactic acid has been proposed as an indicator of resuscitation adequacy [25–27], these results could also mean that acidotic patients who required dobutamine administration were inadequately resuscitated during the early trauma care or had a poor response to volume resuscitation [28]. Regarding the latter, Morris et al. [29] reported that approximately one-third of patients developed early posttraumatic AKI resulted from inappropriate resuscitation. Furthermore, it has been reported that hypotension adversely influences the prognosis of AKI in trauma patients [30].

The association between thoracic trauma and AKI that we found has not been previously reported. Although it is not clear how thoracic injuries could increase the odds of AKI after traumatic rhabdomyolysis, there are possible explanations. Thoracic trauma causes a disturbed immunologic response characterized by elevations in proinflammatory cytokines that can result in multiorgan failure [14, 31, 32]. Therefore, it is plausible to posit that severe thoracic injuries induce persistent posttraumatic inflammation, which in turn results in worse outcomes, such as AKI and multiorgan failure.

In this study, mortality was significantly higher in the group of patients who had KDIGO-3 AKI (Fig. 1). This result suggests a biologic gradient between worse renal function and mortality [24, 33] and that the chances of death are proportional to the severity of AKI. Although we did not analyze the predictors of mortality in our series, it is important to mention the study by Stewart et al. [15]. They examined a large cohort of combat casualties to identify risk factors for rhabdomyolysis and its relation to AKI and death. Beyond showing that patients with AKI had a higher mortality risk, they demonstrated that rhabdomyolysis acted as an effect modifier in the pathway between AKI and death.

### Limitations

Our study is not without limitations, and the results should be interpreted in the context of the study design. First, the retrospective nature of the investigation makes it prone to bias and confusion. Second, further selection bias could be introduced as only patients in whom a CK test was performed were included. Third, the etiology of AKI is usually multifactorial, and rhabdomyolysis may explain the development of AKI only in a proportion of critically ill patients. Furthermore, the fact that our patients were those who underwent CK testing could compromise the external validity. Because we included only patients who had a CK test, our results could be pervaded by selection bias. Therefore, our results can only be extrapolated to a small population in which the pretest probability of rhabdomyolysis and AKI is higher than that in other populations. In other words, our results can be extrapolated to those patients in whom clinical awareness of traumatic rhabdomyolysis is greater. Furthermore, despite our regression analysis, there could be persistent differences in some of the variables (i.e., trauma mechanism) among the groups compared, suggesting that residual confounding affected our analysis.

Finally, a key strength of our analysis is the construction of a multivariable model that included known susceptibility and risk factors for AKI such as age, trauma mechanisms, hypoperfusion variables, resuscitation variables and toxic or protector substance administration.

## Conclusion

We found that age, APACHE II score, thoracic trauma, lactic acidosis, and dobutamine administration were independently associated with AKI. Trauma surgeons need to be aware of the increased odds of AKI if one of these factors is identified during the evaluation and treatment of injured patients.

## Data Availability

Data are the property of the authors and can be obtained by contacting the Principal Investigator: Dr. Alberto F. García; e-mail: alberto.garcia@correounivalle.edu.co

## Abbreviations

AIS: Abbreviated injury scale
AKI: Acute kidney injury
APACHE II: Acute physiology and chronic health evaluation
AUC: Area under curve
CI: Confidence intervals
CK: Creatine kinase
ICU: Intensive care unit
IQR: Interquartile range
ISS: Injury severity score
*OR*: Odds ratios
